# A Developed Robust Model and Artificial Intelligence Techniques to Predict Drilling Fluid Density and Equivalent Circulation Density in Real Time

**DOI:** 10.3390/s23146594

**Published:** 2023-07-21

**Authors:** Mohammed Al-Rubaii, Mohammed Al-Shargabi, Bayan Aldahlawi, Dhafer Al-Shehri, Konstantin M. Minaev

**Affiliations:** 1 Department of Petroleum Engineering, King Fahd University of Petroleum & Minerals, Dhahran 31261, Saudi Arabia; g200453260@kfupm.edu.sa (M.A.-R.); bayan.aldahlawi@kfupm.edu.sa (B.A.); 2 School of Earth Sciences & Engineering, Tomsk Polytechnic University, Lenin Avenue, Tomsk 634050, Russia; al_shargabi@tpu.ru (M.A.-S.); minaevkm@tpu.ru (K.M.M.)

**Keywords:** equivalent circulating density, mud weight, artificial intelligence, drilling efficiency, support vector machine, artificial neural network, decision tree

## Abstract

When drilling deep wells, it is important to regulate the formation pressure and prevent kicks. This is achieved by controlling the equivalent circulation density (ECD), which becomes crucial in high-pressure and high-temperature wells. ECD is particularly important in formations where the pore pressure and fracture pressure are close to each other (narrow windows). However, the current methods for measuring ECD using downhole sensors can be expensive and limited by operational constraints such as high pressure and temperature. Therefore, to overcome this challenge, two novel models named ECD_effc.m_ and MW_effc.m_ were developed to predict ECD and mud weight (MW) from surface-drilling parameters, including standpipe pressure, rate of penetration, drill string rotation, and mud properties. In addition, by utilizing an artificial neural network (ANN) and a support vector machine (SVM), ECD was estimated with a correlation coefficient of 0.9947 and an average absolute percentage error of 0.23%. Meanwhile, a decision tree (DT) was employed to estimate MW with a correlation coefficient of 0.9353 and an average absolute percentage error of 1.66%. The two novel models were compared with artificial intelligence (AI) techniques to evaluate the developed models. The results proved that the two novel models were more accurate with the value obtained from pressure-while-drilling (PWD) tools. These models can be utilized during well design and while drilling operations are in progress to evaluate and monitor the appropriate mud weight and equivalent circulation density to save time and money, by eliminating the need for expensive downhole equipment and commercial software.

## 1. Introduction

Equivalent circulating density (ECD) is a parameter that takes into account the original mud density, the weight of drilling cuttings, and the impact of annular pressure loss in open and cased holes. Several factors affect ECD, including mud weight (MW), hydraulic diameter (the difference between hole diameter and drill string outside diameter), plastic viscosity (PV), yield point (YP), annular velocity ($V_{ann}$), and wellbore geometry factor [1,2,3,4,5]. The calculation of ECD provides important information on the conditions within the borehole and plays a crucial role in drilling operations, particularly in critical gas and oil wells where the drilling mud window is limited [6,7]. Variations in ECD can cause some drilling issues, including poor hole cleaning, reduced rate of penetration (ROP), lost circulation, stuck pipe, and well control incidents. Understanding the concept of ECD and its application is key to achieving optimal well-drilling and rig performance while maintaining safety and environmental standards [8]. Additionally, ECD can serve as a useful tool in planning the trajectory of a wellbore, which is a critical aspect of drilling operations. Although there are various types of wellbore trajectories, directional drilling is now widely used in place of vertical wells to meet the requirements of modern and complex projects while also ensuring that the project is economically viable [2,9,10]. When planning a well path, it is crucial to consider geological factors and material strengths. Moreover, the trajectory of the wellbore can significantly influence hole cleaning, pressure losses, and the management of equivalent circulating density (ECD). To drill an oil well, it is necessary to use drilling fluids that clean, cool, and, most importantly, maintain hydraulic pressure to control the fluids of the geological formation. Monitoring the rheological behavior of the fluid is essential in estimating the hydraulic pressure of the well. The design of the well is heavily influenced by these factors [11]. In critical formations, ECD is utilized to manage the formation pressure and avoid influxes. The existing techniques for computing ECD in oilfields mainly depend on expensive downhole sensors that offer real-time measurements of ECD. However, many of these instruments have limitations in their downhole operation, including high-pressure and high-temperature conditions [10,11]. As downhole ECD tools are expensive and mathematical models are often inaccurate, predicting ECD from drilling parameters has become a new area of focus in drilling engineering. With the aid of advanced computing power, machine-learning techniques can achieve higher prediction accuracy than conventional and statistical models. The accurate calculation of ECD is crucial in drilling and completing an oil well, particularly in deep water, horizontal well sections, or depleted reservoirs, due to its sensitivity. Errors in ECD calculations can lead to disastrous consequences. Annular frictional pressure loss (AFPL) is the influencer of pressure loss in traditional ECD predictions and has garnered increased attention in the literature [10,11,12]. In drilling and completion operations, the impact of the AFPL on the ECD is significant because it provides the total pressure loss at the wellbore. Other factors that influence ECD include the presence of drill cuttings in the wellbore and the depth and diameter of the wellbore. The surge pressure is also controlled during drilling to ensure safe operations and speed up pipe tripping [13,14]. Controlling the well pressure and ECD is a critical aspect of drilling horizontal wells. Precise and frequent measurement of the rheological parameters of the drilling fluid is crucial for effective hydraulic control. Moreover, intelligent drilling, which utilizes information on the drilling fluid to create an optimization model for the ROP, is crucial. Proper drilling fluid designs can enhance drilling efficiency and minimize incidents. Nevertheless, laboratory testing is still the primary method used to determine the drilling fluid’s qualities [13,14].

Real-time measurement of drilling fluid characteristics is crucial for drilling engineering to ensure efficient decision making and the optimization of drilling fluid performance. Failure to identify the properties of the drilling fluid and react in time can result in slower rates of penetration, accidents, and significant financial losses. Laboratory testing alone is not sufficient because it impedes the optimization of drilling fluid performance in real-time conditions. Maintaining optimum mud pressure throughout all operations is one of the prerequisites and techniques to reduce failures and unproductive time, particularly in drilling operations where issues tend to arise more frequently [15]. Due to its potential to cause major drilling issues, ECD management is one of the most critical factors, and optimizing the relevant parameters is essential. When designing and drilling extended-reach wells, managing ECD is a vital consideration. High ECD can cause significant drilling complications, making it crucial to manage this factor carefully [15]. A major drilling issue, such as borehole instability, is also brought on by ECD variation, which is followed by the repetitive creation and erosion of the cuttings deposit bed in drilling extended-reach wells. ECD may swing between high and low readings during drilling and circulation. Lowering the penetration rate or cutting the circulation duration can also reduce ECD volatility. High ECD was caused by insufficient mud circulation, especially when there was a high penetration rate, even though long circulation times caused ECD to fluctuate. Accurate ECD may result from knowledge of the borehole condition [15,16,17]. Many studies have been conducted to predict borehole conditions. Zuo et al. developed a new model to characterize downhole reservoir fluid by decontaminating the effect of oil-based mud [18]. In addition, Gonzalez et al. also showed how to estimate viscosity and density using mechanical oscillators based on tuning forks in various scenarios [19]. The logging-while-drilling (LWD) acoustic and formation pressure tools have been used by Freitag et al. to predict the pore pressure and then discuss the gathering of seismic data while drilling [20]. A real-time pressure monitoring solution was provided with an integration technique of LWD and look-ahead vertical seismic profile (VSP) to drill and complete well LD10-C safely [21]. Alkinani et al. predicted ECD prior to drilling by using an artificial neural network (ANN) [22]. Five efficient artificial intelligent models, including Bayesian ridge regression (BRR), K-nearest neighbors (KNN), support vector machine (SVM), decision tree (DT), and adaptive boosting regressor with decision tree (ABR-DT), were proposed for estimating mud weight based on a databank of 817 data points from five wells in the South Pars gas field [23]. Wang et al. proposed research on the application of the ensemble gradient boost decision tree (GBDT) to develop a robust model that can be used to precisely predict the occurrence of lost circulation [24]. Table 1 provides a summary of the other relevant literature related to borehole prediction.

More importantly, ANNs have been employed in some studies to predict rheological properties. As an example, Elkatatny et al. constructed a mathematical model to predict the properties by only using mud density, marsh funnel, and solid content [35]. Elkatatny also used three previous estimations by developing an empirical equation based on KCl-polymer measurements [36]. On the other hand, Gomaa et al. constructed an empirical model that is suitable for ultradeep gas well drilling [7]. Alkinani et al. utilized the ANN model to predict ECD before drilling [37]. In addition to a prior ANN model, Gamal et al. combined robotic tools with an adaptive neuro-fuzzy interference system (ANFIS) model to predict ECD by only using the surface-drilling parameters [38]. Moreover, Ahmadi (2016) used the least square support vector machine (LSSVM) and ANFIS to predict rheological fluid at high-pressure and high-temperature (HPHT) conditions [39].

Other computer intelligences, such as SVM, random forest (RF), and functional network (FN), have been applied by Alsaihati et al. to predict ECD in high-pressure and high-temperature wells [40]. Rahmati and Tatar estimated the density of drilling fluid by using the radial basis function (RBF) under HPHT conditions [41]. Then, Xianming organized the well pressure and ECD in real-time correction by analyzing some drilling parameters [42]. Table 2 shows several studies that utilized artificial intelligence as a prediction tool.

**Figure 1:**
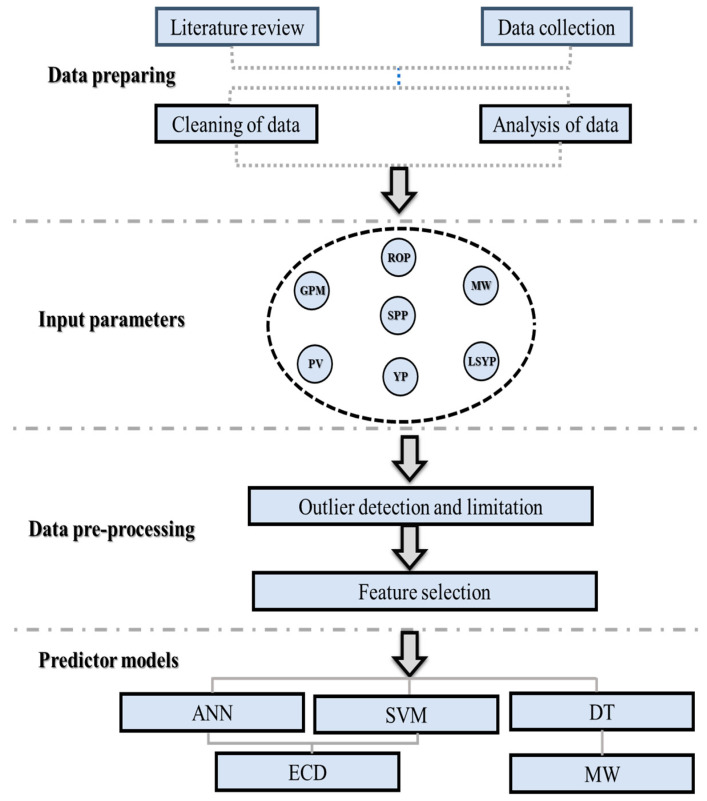
Flowchart of the work with the utilized AI to predict the ECD and MW.

The findings presented in this study demonstrate the capability of the AI model to predict wellbore conditions in real time. Moreover, the reviewed literature has shown that various models have been developed and used to predict mud weight and equivalent circulating density. The Alsaihati [40], Zheng [29], and Xianming [42] models were among those used for ECD prediction, while [23,27,33] were used for MW prediction. However, the reviewed studies did not consider several critical factors that significantly affect drilling operations. These factors include cuttings features, drilling mechanical parameters, well trajectory profiles (which contain inclinations and azimuths), and fluid rheological properties, along with calculated cuttings slip and annular velocities. While some expensive tools are available for measuring these parameters, they have operating limitations such as pressure, temperature, and tool failures. More importantly, the literature review also revealed significant discrepancies between actual drilling hydraulic values and those predicted by previously accepted mathematical equations. To improve the accuracy of drilling hydraulic calculations, other factors, such as pipe eccentricity, wellbore roughness, pressure and temperature, and pipe rotation speed, can be further improved by optimizing the input parameters utilized. Therefore, the novelty of this paper lies in the development of novel models for calculating the equivalent circulating density and the modified mud weight effective. The ECD model takes into consideration parameters such as standpipe pressure (SPP), rate of penetration (ROP), drill string rotation (RPM), mud properties including the modified PV, YP, and low shear yield point (LSYP), angles of borehole and azimuth, modified average cuttings concentration in an annulus, modified hole geometry factor, and other factors. Moreover, the modified mud weight effective model considers the circulation and rotation influence, modified average cutting concentration in an annulus, and the modified hole geometry factor. More importantly, to enhance the model’s performance, the study employs novel methodologies by utilizing artificial neural networks (ANNs) in conjunction with support vector machines (SVMs) and decision trees (DTs). The study aims to estimate ECD using both ANN and SVM models, and predict mud weight using DT. The accuracy of these models has been validated using actual data to confirm their reliability in real-time drilling operations. The novel models for ECD and modified mud weight effective, in conjunction with the use of ANN, SVM, and DT models, represent significant advancements in the field and have the potential to improve the safety and efficiency of drilling operations. The flowchart of the work with the utilized AI to predict the ECD and MW is shown in Figure 1.

### 1.1. Drilling Fluid Rheology and Hydraulic

Rheology and fluid hydraulics are important aspects of drilling operations. Rheology refers to the study of the flow and deformation of materials, including drilling fluids, and hydraulics refers to the study of the movement of fluids, including mud, in the drill table system, which utilizes pipes, valves, and pumps to circulate drilling mud, remove cuttings, cool drill bits, and lubricate the drill string. The rheology of the drilling fluid affects its ability to transfer pressure to the wellbore walls, casing efficiency, wellbore wall stability, and pump performance. The rheological properties of drilling fluid, such as PV, YP, gel strength, and density, can vary with composition and temperature. As an example, if you raise the PV of a fluid, it will cause several effects. These include an increase in the ECD, surge and swab pressure, and the likelihood of differential sticking due to more solids in the fluid. Furthermore, increasing the plastic viscosity will result in a reduction in the rate of penetration due to bad hole cleaning [43,44]. Fluid hydraulics includes the study of fluid pressure, flow, and velocity in the drill table system. The hydraulic properties of the drilling fluid affect the efficiency of various operations, such as mud flushing, drilling, and circulation. For example, properly tuned hydraulics can prevent blockages and ensure efficient waste removal. Thus, mud rheology and hydraulics are important aspects of drilling operations that need to be considered when planning and executing drilling operations [35].

### 1.2. Sensors of Technological Process Parameters ECD and MW during Drilling

Drilling rigs are complex machines that require a variety of sensors to monitor and control the drilling process. These sensors provide real-time data on different aspects of the drilling process, including hole cleaning, drilling speed, and tool performance, which are essential for efficient and safe operations (see Table 1) [45]. Moreover, the implementation of software systems by service companies, operators, and rig contractors has transformed the capture of drilling and well-service operations and equipment data. Through electronic data capture, real-time data are readily available and provide significant value to the industry. The availability of real-time data empowers asset team members to make informed decisions promptly, resulting in more profitable wells for the operator. Surface parameters are fundamental to drilling and well-service operations, and the following parameters are commonly measured: hookload, weight on bit (WOB), ROP, Kelly or stand height (pressure), surface torque, revolutions per minute, pump pressure, pump flow rate (GPM), pump speed, and pit volumes. The accurate measurement and analysis of these parameters are crucial in optimizing drilling and well-service operations. One important sensor used in drilling operations is the measurement-while-drilling (MWD) sensor. MWD sensors collect data on downhole parameters, such as inclination, azimuth, and toolface orientation. These data are transmitted to the surface, allowing drillers to make informed decisions about the drilling process and optimize hole cleaning. Another important sensor is the WOB sensor, which measures the force applied to the drill bit during drilling. This information helps operators optimize drilling parameters, such as drilling speed and bit rotation, ensuring efficient hole cleaning and minimizing the risk of bit damage or stuck pipe. The logging-while-drilling (LWD) sensor provides real-time measurements of formation properties, such as resistivity, porosity, and density [46]. These data are used to evaluate the reservoir, optimize drilling parameters, and monitor hole-cleaning efficiency. The rate of penetration (ROP) sensor measures the speed at which the drill bit penetrates the formation [45,46]. By monitoring ROP, drillers can optimize drilling parameters, such as weight-on-bit and rotational speed, to maximize hole cleaning and drilling efficiency. Mud weight sensors measure the density of the drilling fluid, or mud, which is critical for maintaining wellbore stability and efficient hole cleaning. By monitoring mud weight, operators can make necessary adjustments to the drilling fluid properties, ensuring optimal drilling conditions. All of these sensors work together to provide a comprehensive picture of the drilling process, allowing operators to make data-driven decisions that optimize drilling performance and ensure effective hole cleaning. The data collected from these sensors are transmitted to drilling control centers, where they are analyzed in real time to make adjustments to drilling parameters and ensure safe operations. Moreover, the ECD and mud weight MW are crucial for safe and efficient drilling operations. The ECD sensor measures the density of the drilling fluid or mud during circulation. It takes into account the weight of the mud, the pressure drop across the bit, and the velocity of the fluid. The ECD value is critical for maintaining wellbore stability, as excessive ECD can cause formation damage or even lead to wellbore collapse [6]. The MW sensor measures the density of the drilling fluid or mud in the mud pit. It provides an indication of the mud weight being used and is critical for maintaining wellbore stability and efficient hole cleaning. If the mud weight is too low, it may not be able to carry cuttings out of the borehole, leading to blockages and reduced drilling efficiency. If the mud weight is too high, it may cause formation damage or lead to lost circulation. By monitoring these parameters, drilling operators can ensure that the borehole is being drilled safely and efficiently. The data collected from these sensors are transmitted to drilling control centers, where they are analyzed in real time to make adjustments to drilling parameters and ensure optimal drilling conditions [45,46]. An overview of the kinds of information typically gathered by surface and downhole sensors is shown in Table 3.

However, the quality of the data produced by the rig sensors or downhole sensors has a considerable influence on the dependability and accuracy of real-time drilling conditions. Conventional sensors may not always be sufficient to deliver the data necessary to run some models in real time, which can impede drilling teams’ ability to immediately modify courses to prevent or decrease hole-cleaning concerns [47,48]. Drilling parameters, which are modified in real time, are crucial. These modifications have an impact on surface operating parameters such as ROP, flow rate, RPM, and WOB. There may or may not be a distinction between real-time and non-real-time parameters, depending on how real-time is defined.

More significantly, it is essential to manage the wellbore pressure, control the formation pressure, and prevent kicks when drilling deep wells. ECD is particularly essential in formations when the pore pressure and fracture pressure are narrow windows. However, the existing methods for detecting ECD utilizing downhole sensors can be costly and constrained by operating restrictions such as high pressure and temperature. Therefore, to address this difficulty, the following section discusses two unique models named ECD_effc.m_ and MW_effc.m_ with techniques designed to forecast ECD and MW from surface-drilling data, including standpipe pressure, rate of penetration, drill string rotation, and mud characteristics.

## 2. Methodology

The methodology discusses the data analysis, feature selection, splitting data, model selection, which are ANN, SVM, and DT, which is a simple and efficient algorithm for large datasets and is easy to visualize, and the quality control and quality assurance (Qc & QA) of the models. This section provides a detailed explanation of the selection models, real-time factors, and workflow involved in applying computer-based intelligence to predict ECD and MW.

### 2.1. Artificial Neural Network (ANN)

**Figure 2:**
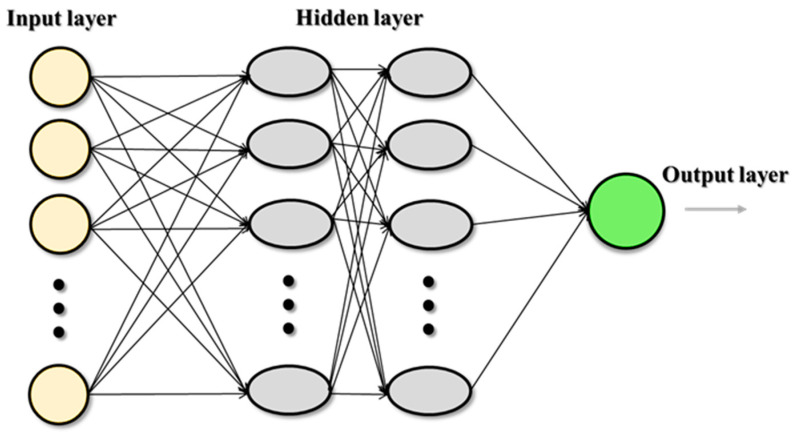
General schematic diagram of ANN.

ANNs have the capacity to estimate complicated nonlinear functions that exist between input and output parameters, as claimed by Fausett in 1994 [49]. The three major parts of the ANN are a learning algorithm, a transfer function, and a network design with at least three layers (input, hidden, and output). ANNs are made up of simple processing units called neurons. Each layer of the hidden structure, which may have one or more layers, is related to the others by weights. The change in these weights between the layers affects the network’s performance. The ANN is first trained by feeding data into its input layer, then via any necessary hidden layers, and, ultimately, to its output layer. The output layer compares the data to the real data. Moreover, the model training should continue for the full dataset until the average error is below a predetermined threshold. The process of updating the specific weights and biases between each layer link in the model during each epoch is known as using the difference between actual and forecasted data [35]. The ANN approach was chosen for this study above other artificial intelligence network techniques because it can generate extremely precise mathematical equations. Additionally, the ANN-developed models are relevant on the rig site since they can be utilized by everyone without the requirement for specialized software [49]. Figure 2 shows a general schematic diagram of the ANN.

### 2.2. Support Vector Machine (SVM)

**Figure 3:**
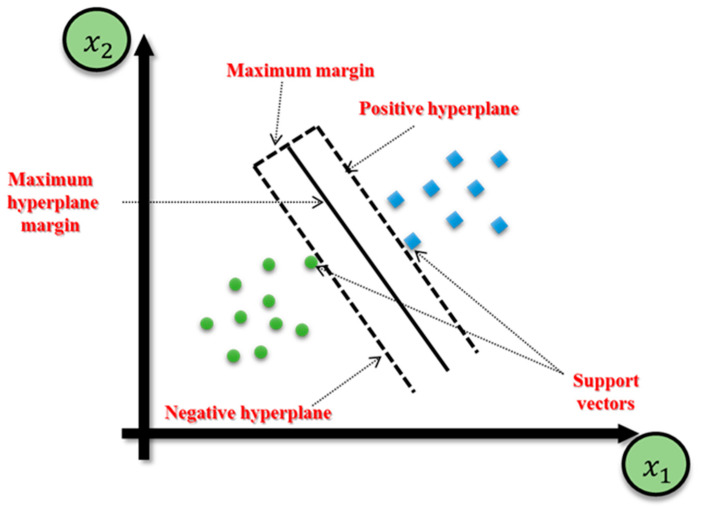
The execution of an SVM classifier on a dataset containing two classes and two features (linear SVR).

The first iteration of SVM was developed at AT&T Bell Labs, and, in 1997, H. Drucker and colleagues introduced support vector regression (SVR). SVR shares many similarities with SVM, and its structure is designed to estimate a function that maps input to a numerical output [50]. In comparing the two, SVM does not penalize points far from the hyperplane in classification problems if the class is predicted accurately, while SVR penalizes all points outside the margin to obtain a function that closely approximates target points. SVR only considers errors greater than a specified threshold. Generally, in classification problems with nonlinearly separable data, kernel functions are used to transform the data into a higher-dimensional feature space, enabling linear separation. In regression scenarios, kernelization is applied for nonlinear SVR [51,52,53,54]. Figure 3 shows the execution of an SVM classifier on a dataset containing two classes and two features (linear SVR).

### 2.3. Decision Tree (DT)

DTs are predictive modelling tools used in a number of different domains. They are typically constructed using an algorithmic approach, which defines ways of partitioning a set of data based on different conditions [55]. It is one of the most widely used and practical methods of supervised learning. Decision trees are nonparametric teacher-assisted learning methods used for both classification and regression tasks [56]. The goal is to create a model that predicts the target feature by learning simple decision rules derived from the characteristics of the data [56]. Establishing a stopping condition that stops the data splitting procedure is crucial to avoiding overfitting in decision trees. The internal node serves as the intermediary in this process, which starts at the root node. The stopping condition can be specified in a variety of ways, such as defining a maximum depth for each leaf, a minimum number of samples required to split an internal node, and a minimum number of samples needed to split a leaf node, and restricting the number of features that are taken into account when looking for the best split. The decision tree’s complexity is decreased, and the risk of overfitting is minimized by putting these constraints in place. The tree will keep splitting if there is no stopping condition, creating a complicated decision tree [23].

### 2.4. Field Data Description

The primary objective of the study is to achieve real-time prediction of mud weight (MW) and equivalent circulating density (ECD) during drilling operations with the aid of artificial intelligence (AI). This approach is intended to enhance drilling efficiency by providing accurate and reliable results. To achieve this goal, the study utilized a large dataset, which includes 4371 records for ECD and 33,588 records for MW from offshore gas Deviated Well-A, offshore oil Deviated Well-B, and horizontal oil Well-C. The interval between X3000 and X4200 ft was chosen for the application of AI and the two novel models due to its abundance of accurate and essential data. This interval was deemed sufficient because it contained all the necessary information without any missing data. By selecting this specific interval, the study was able to ensure the accuracy and reliability of their analysis while optimizing the use of resources and time. This approach allowed the researchers to focus on the most relevant and significant data, thereby enhancing the overall quality and validity of their findings. More importantly, the study considered various parameters to determine the density of drilling fluid, including rheological properties such as plastic viscosity and yield point, which were assessed using a rheometer at 48 °C and standard atmospheric pressure. Additionally, Marsh funnel viscosity was evaluated using a Marsh funnel at room temperature and ambient pressure, and the percentage of solids was measured using a mud retort to evaporate the liquid phase and gather the remaining solids. Other vital parameters, such as mud pump flow rate, rate of penetration, and standpipe pressure, were also recorded.

The dataset was split into a training set (80%) and a testing set (20%), with the random state set to 42 to ensure consistency in the training and testing sets across different executions. The split ratio of 80:20 was chosen because it is the most common ratio, and, since hyperparameter tuning was performed, a validation set was not deemed necessary.

The study utilized three models, namely, decision tree (DT), artificial neural network (ANN), and support vector machine (SVM), implemented in Python to predict MW and ECD. The description of the three models to predict the ECD and MW is described as follows:

**a.**  **Equivalent circulating density (ECD) prediction**

For ECD prediction, the correlation between ECD-PWD (pound cubic feet (PCF)) and several features, such as SPP (psi), LSYP, and GPM, was analyzed. ECD-PWD (PCF) had the strongest positive correlation with GPM (0.951726), and the other features also had a positive correlation with ECD-PWD (PCF), with values larger than 0.7.

**b.**  **Mud weight (MW) prediction**

For mud weight prediction, the dataset was analyzed to find the correlation between the mud density suction and GPM. It was found that the mud density suction has a positive correlation with depth (0.864299). The dataset was split into an 80% training set and a 20% testing set (not random).

### 2.5. Validation of the Developed Correlations

**Figure 4:**
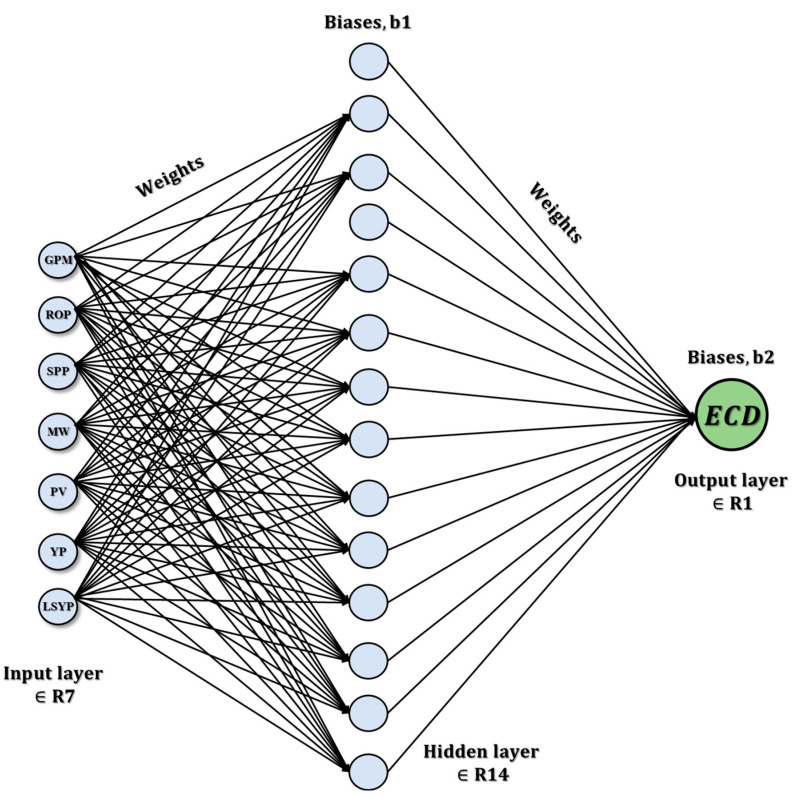
Construction concept using the ANN model.

The study aims to present a real-time prediction of MW and ECD using intelligence retrieval or AI to maximize drilling efficiency. Figure 4 shows the workflow of the application of computer-based intelligence to predict ECD and MW. The collected data required for this research were analyzed, and the features in the input were selected. The correlation between the estimated values and the real values was calculated.

Data analysis, feature selection, data splitting, model selection, and Qc & QA of the models are covered in the process. There are many factors that affect the efficiency of machine-learning models for predicting the success of learning, including the following:

- The size of data. The more data added, the higher the accuracy and trust in the results. The size of the ECD data is 4371, and the size of the mud weight data is 33,588.
- Impacts of predictability depend on feature/input selection in the data. The variables with a high correlation coefficient value were selected, and the variables with a lower correlation coefficient value were dropped. For the ECD-PWD (PCF) target, GPM (gal/min), SPP (psi), and ROP (ft/h) have strong positive correlations of 0.864299, 0.803474, and 0.729802, respectively, whereas mud density suction with depth has a strong correlation of 0.574443.
- Choosing the best machine-learning algorithm that fits the AI Project. The MW did not work with ANN and SVM, while estimating the ECD had a good result by using ANN and SVM models; however, real-time MW prediction worked successfully with the DT.
- The coefficient of determination (R^2^), mean squared error (MSE), and mean absolute error (MAE) statistical metrics were used to evaluate ML prediction performance in this work.

#### Data Analysis and Feature Selection

The study found that mud density suction has a positive correlation of 0.864299 with GPM for MW prediction. This indicates that, as the mud density suction increases, the GPM also increases, resulting in a higher mud weight. Similarly, for ECD prediction, the study found that ECD-PWD (PCF) has the strongest positive correlation of 0.951726 with GPM. This indicates that, as the GPM increases, the ECD-PWD (PCF) also increases, resulting in a higher equivalent circulating density. The features selected for MW prediction are GPM, ROP, PV, YP, and LSYP, and the target variable is MW. The features selected for ECD prediction are GPM, ROP (ft/h), SPP (psi), MW (PCF), PV, YP, and LSYP, and the target variable is ECD-PWD (PCF). Figure 4 shows that the ANN diagram for ECD represents a distributed processing system consisting of neurons that are connected nodes that receive input, process the data, and provide an output.

The Sklearn library [57] has been used for the decision tree regression model by applying MW prediction and hyperparameters, including tuning the max depth to 7 to return the maximum depth of the tree and setting the criterion to the squared error for the mean squared error, and is used to measure the quality of a split by minimizing the L_2_ loss using the mean of each terminal node. The coefficient of determination (R^2^) value is 0.94, and the accuracy MW is 94%. The MSE value is 0.08.

**ANN model**

The TensorFlow library [58] has been used for ANNs with hyperparameters consisting of one input layer with seven neurons and a rectified linear unit (ReLU) activation, one hidden layer with fourteen neurons and a ReLU activation, and one output layer with one neuron. The Adam algorithm has been implemented for the optimizer to update network weights during training. The accuracy of the ANN model is 99.47%.

**Support vector machine** **model**

The Sklearn library [57] has been used for SVM, with hyperparameter tuning to rbf kernel, C to 100.0, and epsilon to 1. The R^2^ value is 0.99, and the MSE value is 2.02. The prediction of the ECD-PWD (PCF) plot is precise and reaches 99.2% accuracy.

**Qc & QA**

The best model is selected by testing the dataset and applying different models. Hyperparameters have been tuned, and the models have been tested many times to ensure the models’ accuracy. Its correctness has been checked by measuring the correlation between the calculated and real data [59].

### 2.6. Mathematical Development of the Model for the ECD_effc.m_ and MW_effc.m_

The two novel developed models ${MW}_{eff.m}$ and ${ECD}_{eff.M}$ were obtained starting from the effective mud weight ${(MW}_{eff}$) calculated using Equation (1) [60], where MW is the static drilling fluid density (lb/cf) and $CCA({CCA}_{API})$ is the cuttings concentration in an annulus as defined by Equation (2) [2,61].

(1) $${MW}_{eff}=MW\cdot {CCA}_{API}+MW$$

(2) $${CCA}_{API}=\frac{ROP{\cdot OH}^{2}}{1471\cdot GPM\cdot TR}$$

where *ROP* denotes the rate of penetration (ft/h), *OH* is the diameter of the hole (inch), 1471 is the conversion factor to convert a *GPM* into gallons per minute, *GPM* is the flow rate of the mud pump (gal/min), and *TR* is the transport ratio, which can be substituted with 0.55 in accordance with [61]. Furthermore, the *ECD* is determined based on the ${MW}_{eff}$ in real time (PCF). To be precise, Equation (3) can be utilized to calculate the *ECD* [60].

(3) $$ECD={MW}_{eff}+\left(\left(\frac{0.085}{OH-{OD}_{pipe}}\right)\cdot \left(YP+\frac{PV\;Vann}{300(OH-{OD}_{pipe})}\right)\right)\cdot 7.481$$

where OD is the outer diameter of the drill pipe (inch), PV is the plastic viscosity (PV = *R*600 − *R*300) (*CP*), YP is the yield point (YP = *R*300 − PV*)* (lb/100 sqft), and Vann is the annular velocity of the drilling fluid (ft/min) as defined by Equation (4).

Equation (6) is used to determine the $V_{ann}$ [62]:

(4) $$V_{ann}=\frac{24.5GPM}{{OH}^{2}-{{OD}_{pipe}}^{2}}={V_{cr}+V}_{sc}$$

where $V_{cr}$ is the cutting rise velocity (ft/min) and $V_{sc}$ is the cutting slip velocity with the effect of the rate of penetration (ft/min).

The LSYP is a crucial parameter that indicates the minimum force required to initiate fluid movement in the wellbore, which equals LSYP = 2R3 − R6 [5]. It is vital to ensure that the drilling fluid can effectively suspend and transport cuttings out of the wellbore. Consequently, it is essential to take LSYP into account along with PV and YP to ensure that the drilling fluid system is optimized for efficient and safe drilling operations. This will help prevent operational challenges and ensure that the drilling process is carried out smoothly and effectively. From Equation (3), ECD can be modified as follows based on PV and YP (Equations (5) and (6)) [63]:

(5) $${PV}_{m}=\left(R600-LSYP\right)-\left(R300-LSYP\right)$$

(6) $${YP}_{m}=2\left(R300-LSYP\right)-(R600-LSYP)$$

where *R*600 represents the Fann reading viscometer at 600 RPM, and *R*300 represents the Fann reading viscometer at 300 RPM. More importantly, the consistency factor (cP) and the flow behavior index, represented by *k* and *n,* respectively, are of utmost importance. These values need to be optimized for specific drilling conditions to ensure that the borehole is cleaned efficiently. If the values of *k* and *n* are not carefully monitored, the drilling fluid may struggle to suspend and transport cuttings out of the wellbore, leading to significant operational challenges. It is therefore crucial to keep a close watch and adjust the values of *k* and *n* to ensure that the drilling fluid system is functioning optimally. Therefore, the $k_{m}$ and $n_{m}$ factors can be modified and obtained from Equations (7) and (8) to consider the viscometer reading at 600 RPM, 300 RPM, 6 RPM, and 3 RPM of ${PV}_{m}$, ${YP}_{m}$, and *LSYP* [5].

(7) $$k=\left(\left(PV+YP\right)\right){\left(510\right)}^{1-n}=k_{m}=\left(\left({PV}_{m}+{YP}_{m}\right)-(LSYP\right){)\left(510\right)}^{-n_{m}}$$

(8) $$n=3.32\log\left(\frac{\left(2PV+YP\right)}{\left(PV+YP\right)}\right)=n_{m}=3.32\log\left(\frac{\left(2{PV}_{m}+{YP}_{m}\right)-(2R3-R6)}{\left({PV}_{m}+{YP}_{m}\right)-(2R3-R6)}\right)$$

where *R*6 represents the reading viscometer at 6 RPM, and R3 represents the reading viscometer at 3 RPM. The $V_{ann}$ in Equation (4) represents the original annular mud velocity applied in the vertical hole section alone; $V_{ann}$ is given as a function of *GPM*, *OH*, and *OD*. The modified annular velocity ($V_{ann.m}$), as defined in Equation (9), depends on the weight and flow rate of the drilling fluid, the size of the drilled hole, the outer diameter of the drill pipe, the rate of penetration, the rotation of the drill string, the plastic viscosity, the yield point, the viscometer readings at 600, 300, 3, and 6 rpm, the wellbore inclination, and the azimuthal directions [63].

(9) $$V_{ann.m}=\left(\frac{24.5\cdot GPM}{{OH}^{2}-{{OD}_{pipe}}^{2}}cos\left(\alpha \right)+\left(\frac{60}{\left(1-{\left(\frac{{OD}_{pipe}}{OH}\right)}^{2}\right)\left(0.64+\frac{18.2}{ROP}\right)}+\frac{ROP\left(OH^{2}\right)}{60\left(OH^{2}-{{OD}_{pipe}}^{2}\right)}\right)\sin\left(\beta \right)\right)\;-\frac{175\left(d_{cm}\right){\left(\frac{Wc}{7.481}-\frac{{MW}_{eff}}{7.481}\right)}^{2n_{m}}}{({MW}_{eff}{/7.481)}^{n_{m}}{(\frac{2.4\cdot V_{ann.dp}}{OH-{OD}_{pipe}}\left(\frac{2n_{m}+1}{3n_{m}}\right)\left(\frac{200K_{m}\left(OH-{OD}_{pipe}\right)}{V_{ann.dp}}\right)}^{n_{m}}}$$

where Wc is the cutting density, which can be obtained as $Wc=\left({MW}_{eff}\;CCA+{MW}_{eff}\;\right)+\left(1-CCA\right){MW}_{eff}$ according to [47,64]; α is the borehole angle (degrees); β is the azimuth angle (degrees); $d_{cm}$ is the modified cutting diameter, which can be obtained as follows: $d_{cm}=0.2\cdot \frac{ROP}{RPM+xGPM}$, in accordance with [60], where the specification of the mud motor, such as the revolution per gallon ratio (*x*) and GPM of the mud pump flow rate, can be calculated while determining $d_{cm}$; and $V_{ann.dp}$ is the annular velocity across the drill pipe and can be calculated as follows: $V_{ann.dp}=\frac{24.5\left(GPM\right)}{{OH}^{2}-{{OD}_{pipe}}^{2}}$, in accordance with [5,8,60].

More importantly, Newit developed a more precise model for steady-state lifting of materials in a vertical tube by utilizing Equation (2), which can be referenced in Equation (10) [61]. Mitchell presented evidence that an annular concentration model can be developed by considering both the circulation that occurs after drilling has stopped but before a connection is made and the circulation that occurs after a connection but before drilling resumes. The period referred to as preconnection circulation is also known as later circulation. Equation (11) describes the Mitchell’s cutting concentration in an annulus ${(CCA}_{2}$) [60,61].

(10) $${CCA}_{1}=-\frac{1}{2}\left(\frac{V_{ann.m}}{V_{sa}}-1\right)+{\left(\frac{1}{4}{\left(\frac{V_{ann.m}}{V_{sa}}-1\right)}^{2}+\frac{V_{ann.m}}{V_{sa}}\frac{Vc}{\frac{GPM}{7.48}}\right)}^{0.5}$$

(11) $${CCA}_{2}=\frac{1}{1+\left(1-\frac{OD}{OH}\right)(\frac{V_{ann.m}-V_{sa}}{30})(\frac{1800}{1+ROP}+\frac{V_{sa}}{V_{ann.dc}-V_{sa}}\cdot T_{PC})}$$

where $V_{ann.m}$ is the annular modified velocity of the drilling fluid (ft/min) (Equation (9)); $V_{ann.dc}$ is the annular velocity across the drill collar (ft/min), which can be obtained as $V_{ann.dc}=\frac{24.5(GPM)}{{OH}^{2}-{{OD}_{c}}^{2}}$; $T_{PC}$ is the preconnection circulation time, which indicates the time needed to circulate the cuttings to a height that will prevent them from settling to the bottom of the hole during that connection, which equals $T_{PC}=\frac{V_{sa}}{V_{ann.m}-V_{sa}}\cdot T_{C}$; $T_{C}$ refers to the time for making a connection (min); Vc is the volumetric rate of cutting entering the annulus, which equals $Vc=\frac{ROP\cdot {OH}^{2}}{1100}$ (fpm); and $V_{sa}$ is the average slip velocity of drilling cutting (ft/min), which can be obtained from (12) [60,61]. Moreover, the $V_{sa}$ can be calculated by considering the axial and radial cutting slip velocities with the influence of inclination and azimuth, as mentioned by Azar [65,66] and Robello [67]; therefore, $V_{sa}$= $V_{saa}$ $cos\left(\alpha \right)$ +V_sar_ sin⁡(β), where V_sar_ is the redial cuttings slip velocity (ft/min) and $V_{saa}$ is the axial cutting slip velocity (ft/min).

(12) $$V_{sa}=V_{saa}cos\left(\alpha \right)+V_{\mathrm{sar}}\;\sin(\beta )=\frac{V_{s.m}+Vsc}{2}$$

where $V_{s.m}$ is the average drill cutting slip velocity (ft/min).

Vsc can be obtained from Equation (13). The $V_{s.m}$ can be calculated from Equation (16), which can be modified to present the influence of mud weight and is measured in ft/min and other factors. Moreover, $V_{s.m}$ contains the cutting velocities calculated based on the weight cutting, drilling fluid’s effective viscosity, and rate of penetration [62,68,69,70]. Furthermore, Hopkin demonstrated that the mud weight ($F_{m}$) can affect the slip velocity and developed Equation (16) [71]. Therefore, $V_{s.m}$ can be obtained from Equation (17) by considering the influence of mud weight ($V_{s.mn}$). Furthermore, Equation (17) shows that $V_{s1}\;$ and $V_{s2}$ represent the velocities that are determined by taking into account the effective viscosity, apparent viscosity, weight, and diameter of the cuttings, which are also considered in Equations (14) and (15) (ft/min) [60].

(13) $$V_{sc}=\frac{24.5\;GPM}{OH^{2}-{{OD}_{pipe}}^{2}}-\frac{60}{\left(1-{\left(\frac{{OD}_{pipe}}{OH}\right)}^{2}\right)\left(0.64+\frac{18.16}{ROP}\right)}$$

(14) $$V_{s1}=0.45\;\left(\frac{M_{eff}}{\left(\frac{{MW}_{eff}}{7.481}\;d_{cm}\right)}\right)\;{\left(\left(\frac{36,800\;\frac{{MW}_{eff}}{7.481}\;{d_{cm}}^{3}\;\left(\frac{Wc}{7.481}-\frac{{MW}_{eff}}{7.481}\right)}{{M_{eff}}^{2}}\right)+1\right)}^{0.5}-1)$$

(15) $$V_{s2}=\left(\frac{175\left(d_{cm}\right){\left(\frac{Wc}{7.481}-\frac{{MW}_{eff}}{7.481}\right)}^{0.667}}{{\left(\frac{{MW}_{eff}}{7.481}\right)}^{0.333}Mapp^{0.333}}\right)$$

(16) $$V_{s.m}=V_{sc}\cdot F_{m}=V_{sc}\cdot \left(2.117-\frac{0.1648{MW}_{eff}}{7.481}+0.003681{\left(\frac{{MW}_{eff}}{7.481}\right)}^{2}\right)$$

(17) $$V_{s.m}=\frac{V_{s1}+V_{s2}+V_{sc}}{3}=V_{s.mn}=\left(\frac{V_{s1}+V_{s2}+V_{sc}}{3}\right)\cdot \left(2.117-\frac{0.1648{MW}_{eff}}{7.481}+0.003681{\left(\frac{{MW}_{eff}}{7.481}\right)}^{2}\right)$$

where $M_{eff}$ is the drilling fluid’s effective viscosity, which can be calculated as $M_{eff}={PV}_{m}+{300YP}_{m}\cdot \frac{d_{cm}}{V_{ann.m}}$ and Mapp is the apparent viscosity, which can be calculated as follows: $Mapp={\left(\left(\frac{2.4\cdot V_{ann.dp}}{OH-OD}\left(\frac{2n_{m}+1}{3n_{m}}\right)(\frac{200K_{m}\left(OH-OD\right)}{V_{ann.dp}}\right)\right)}^{n_{m}}$, in accordance with [60]. From Equations (2), (10), and (11), the average ${CCA}_{am}$ can be obtained as shown in Equation (18). Additionally, the new $V_{s.mn}$ can be added to Equations (10) and (11) to obtain a more precise average ${CCA}_{am}$, which contains all the affect parameters and the velocity annular cutting for the drill collar, the drill pipe, and connection time, as follows, in Equation (18) [61].

(18) $${CCA}_{am}=\frac{{CCA}_{API}+{CCA}_{1}+{CCA}_{2}}{3}$$

More importantly, the hole geometry factor, $\alpha_{m}$, is a crucial parameter because it enables the simulation and evaluation of various types of well profiles with different hole sizes and liner diameters (see Equation (19)). This is achieved by utilizing ${CCA}_{am}$, which can be generated while drilling on the wall of the hole size and is referred to as $\alpha_{m.c}$. By modifying $\alpha_{m}$, it is possible to optimize well design and drilling operations to achieve the desired outcomes. Different well profiles and hole sizes can affect the flow and transport of fluids and cuttings, and, hence, it is important to consider the impact of these factors on the drilling process. The hole geometry factor enables engineers and researchers to evaluate and compare different well profiles and drilling scenarios, thereby enabling the selection of the optimal design and drilling parameters for a specific application [72].

(19) $$\alpha_{m.c}=\alpha_{m}+{CCA}_{am}$$

**Figure 5:**
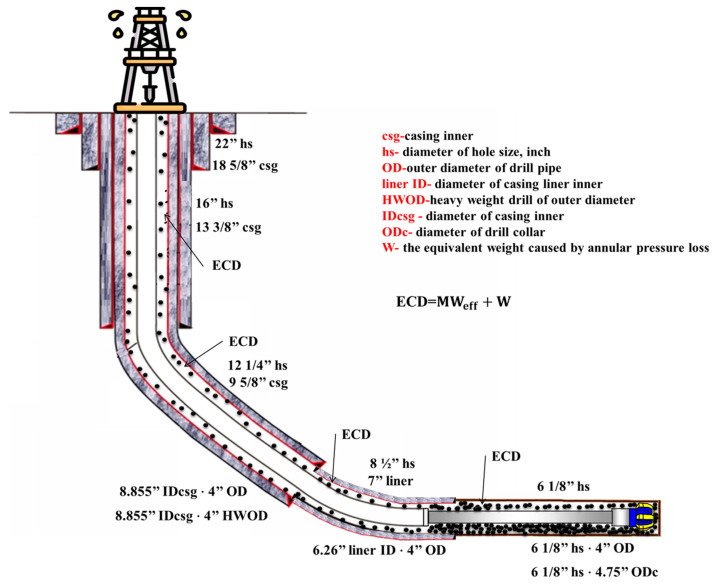
The ECD across different annular area geometries (different geometries).

Furthermore, we consider the ratios of annular areas of the actual hole size and evaluate *ECD* across all the annular areas. The ratios easily show how the cuttings move through all hole sizes and profiles (see Equation (20)). Moreover, according to [62], to consider the *ECD* evaluation through different annular area geometries between hole size, casing inner (${ID}_{csg}$), liner inner diameter (${Line}_{ID}$) size, drill collar (${OD}_{c}$), and drill pipe, Equation (20) shows that all these different diameters influence the ECD (see Figure 5). Additionally, the circulation and rotation influence on the annulus of the hole section (CRF) was considered according to [73]. The *CRF* is a measure of the efficiency of the drilling fluid in transporting cuttings out of the wellbore and preventing accumulation of cuttings based on circulation and rotation influence [73].

(20) $$\frac{1}{\alpha_{m}}=\left(\frac{OH^{2}-O{Dc}^{2}}{OH^{2}}\right)+\left(\frac{{{Line}_{ID}}^{2}-OD^{2}}{OH^{2}}\right)+\left(\frac{ID_{csg}^{2}-OD^{2}}{OH^{2}}\right)+\left(\frac{OH^{2}-OD^{2}}{OH^{2}}\right)$$

(21) $$CRF=\frac{\left(\left(1+GPM\right)-\left(1+RPM\right)\right)}{\left(\left(1+GPM\right)+\left(1+RPM\right)\right)}$$

Equations (19)–(21) can be utilized and added to Equation (1) to obtain the modified mud weight effective ${MW}_{effc.m}$ (see Equation (22)). Ideally, according to [72], $\alpha_{m.c}$ was added to $\;{ECD}_{effc.m}$ and multiplied only by $V_{ann.m}$. Accordingly, the final modified ${ECD}_{effc.m}$ can be obtained from Equation (23).

(22) $${MW}_{effc.m}=\left(MW\cdot {CCA}_{am}\cdot (\frac{\alpha_{m.c}}{CRF})+MW\right)$$

(23) $$\begin{array}{c} {ECD}_{effc.m}={MW}_{effc.m}\\ +((\frac{0.1}{OH-{OD}_{pipe}})\;({YP}_{m}\\ +\frac{{PV}_{m}\left(\alpha_{m.c}\right)\left(V_{ann.m}\right)}{300\left(OH-{OD}_{pipe}\right)}))7.481)\;(\frac{{SPP}_{x}}{{SPP}_{1}})(\frac{{Depth}_{1}}{{Depth}_{x}})) \end{array}$$

where ${SPP}_{x}$ is the current real-time measurement of stand pipe pressure (*SPP*) (psi), ${SPP}_{1}$ is the first *SPP* when *ROP* > 1 or the reading of *SPP* on the last casing shoe depth (psi), ${Depth}_{1}$ is the start measured hole depth (ft), and ${Depth}_{x}$ is the current measured hole depth (ft).

Ideally, ${ECD}_{effc.m}$ takes into account the additional friction pressure loss and incremental depth in directional wells in the annulus and provides a more accurate measure of the pressure and depth exerted on the wellbore and the formation (see Equation (23)). ${MW}_{effc.m}$ takes into account the hydrostatic pressure exerted by the mud on the wellbore and the formation and provides a more accurate measure of the mud weight required to maintain wellbore stability and prevent formation damage (see Equation (22)). The two equations were developed as a novel model that can be used for real-time evaluations and applied in various profiles, such as deviated and horizontal wells, and included other important parameters, such as *LSYP,* to evaluate the hole-cleaning efficiency in deviated, highly deviated, and horizontal laterals while drilling.

## 3. Results and Discussions

In order to maximize drilling efficiency in field applications, this section permits real-time prediction of the above two novel models for *ECD_effc.m_* and *MW_effc.m_* while drilling, combining AI and the suggested drilling control automation. The method offers precise and timely forecasts, enabling the best changes to be made while drilling operations are being conducted. With the help of this technology, drilling teams may increase operational effectiveness and decrease downtime, which will, ultimately, result in cost savings and enhanced drilling performance.

### 3.1. Field Applications Using the Novel Models ECD_effc.m_ and MW_effc.m_

**Figure 6:**
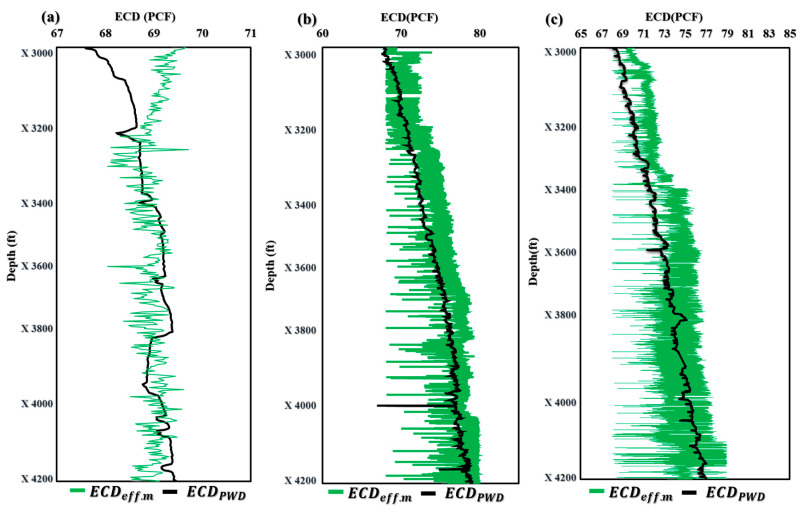
Application of ECD_effc.m_ in offshore gas Deviated Well-A (**a**), offshore oil Deviated Well-B (**b**), and horizontal oil Well-C (**c**).

The application of the novel models ECD_effc.m_ and MW_effc.m_ in maximizing drilling efficiency has been proven in field applications. These models allow drilling crews to make knowledgeable judgements and modify their drilling operations in real time because they provide precise and fast estimates of ECD_effc.m_ and MW_effc.m_. The end effect is greater safety, less downtime, and higher efficiency, which, ultimately, boosts oil and gas drilling and production performance and lowers costs. The effective use of these models in the field demonstrates their potential as useful tools for increasing drilling operations’ efficiency and the sector as a whole. Figure 6a demonstrates that the ECD_effc.m_ model projected an ECD of 69.8 PCF in Well-A at a depth of X3000 ft, but the ECD-PWD was actually measured at 68 PCF. However, the model showed remarkable accuracy in forecasting the ECD at depths ranging from X3296 to X4200 ft. Similar ECD values were predicted by the ECD_effc.m_ model in Figure 6b,c, further proving its applicability and accuracy. These findings shed important light on the effectiveness of the innovative ECD_effc.m_ model and its potential to raise the precision of ECD forecasts made during drilling operations.

**Figure 7:**
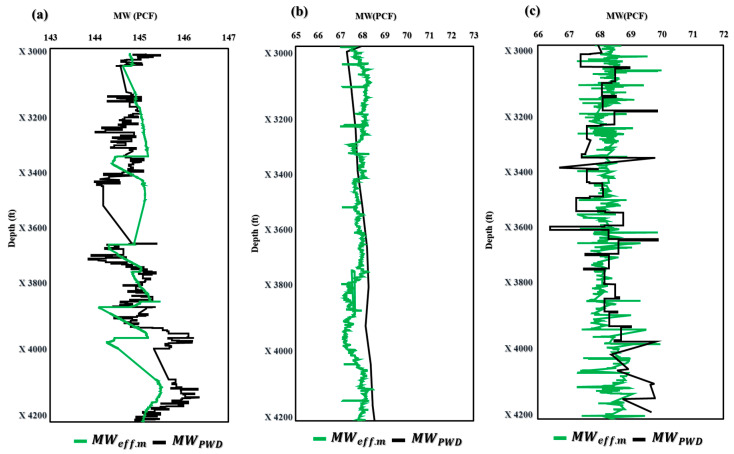
Application of MW_effc.m_ in offshore gas Deviated Well-A (**a**), offshore oil Deviated Well-B (**b**), and horizontal oil Well-C (**c**).

Figure 7 shows the application of the novel model MW_effc.m_ in three wells with different profiles and drilling fluids. Figure 7a illustrates that the novel model for MW_effc.m_ produced similar results, except for depths ranging from X3400 to X3669 ft and X3931 to X3996 ft, which showed less accuracy. However, for wells B and C, the MW_effc.m_ model demonstrated almost identical results (Figure 7b,c). These findings suggest that the MW_effc.m_ model can be a valuable tool for predicting MW in real-time drilling operations, improving efficiency and safety while reducing downtime and costs. Further research and refinement of the model could lead to even greater accuracy and precision in predicting MW, ultimately enhancing performance in the oil and gas industry.

**Figure 8:**
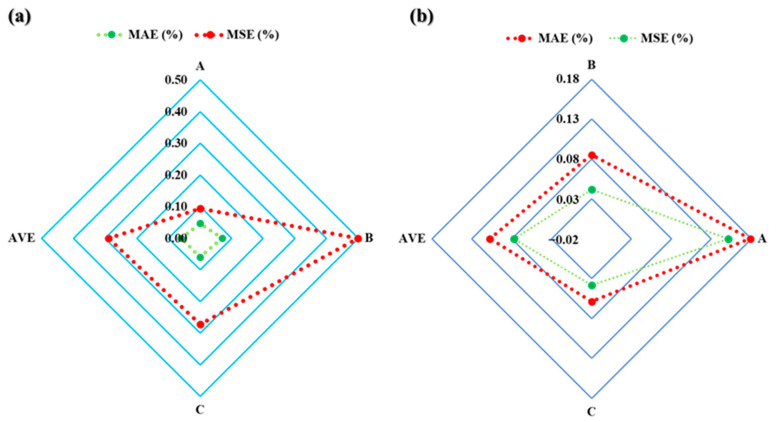
The accuracy statistical measures for the novel models ECD_effc.m_-PWD (**a**) and MW_effc.m_-PWD (**b**).

Figure 8a and Table 4 show that the average accuracy statistical measures for the novel model ECD_eff.m_ were 0.06% and 0.29% for the MAE and MSE, respectively. Moreover, as seen in Table 4 and Figure 8b, the average accuracy statistical measures for the novel model MW_effc.m_ were 0.11% and 0.08% for MAE and MSE, respectively.

### 3.2. AI Applications Using the Novel Models ECD_effc.m_ and MW_effc.m_

**Figure 9:**
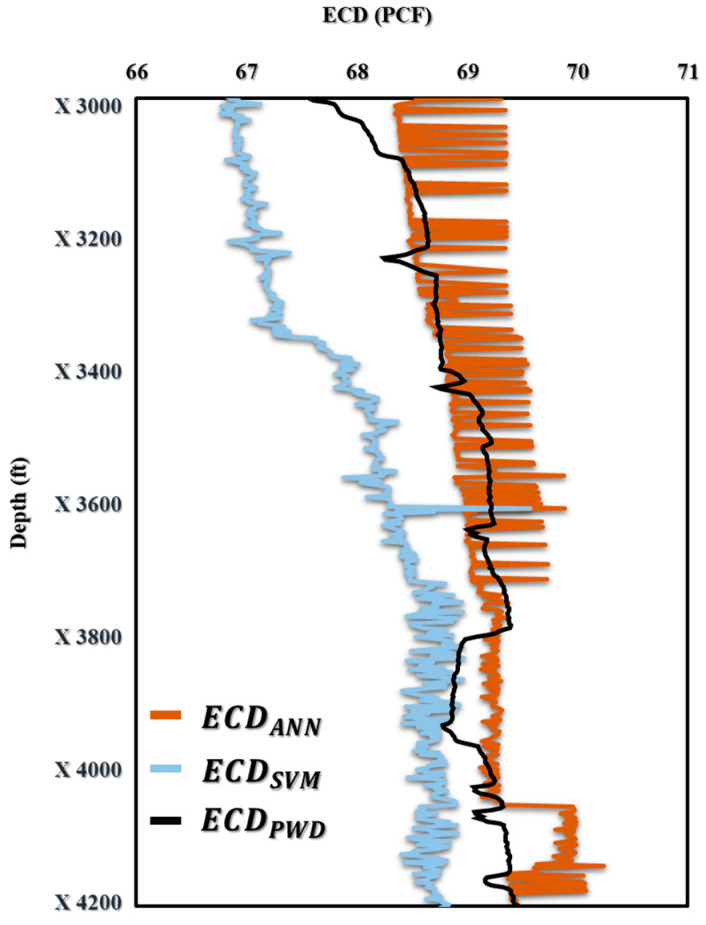
The prediction of ECD (PCF) vs. Depth (ft) for offshore gas Deviated Well-A.

**Figure 10:**
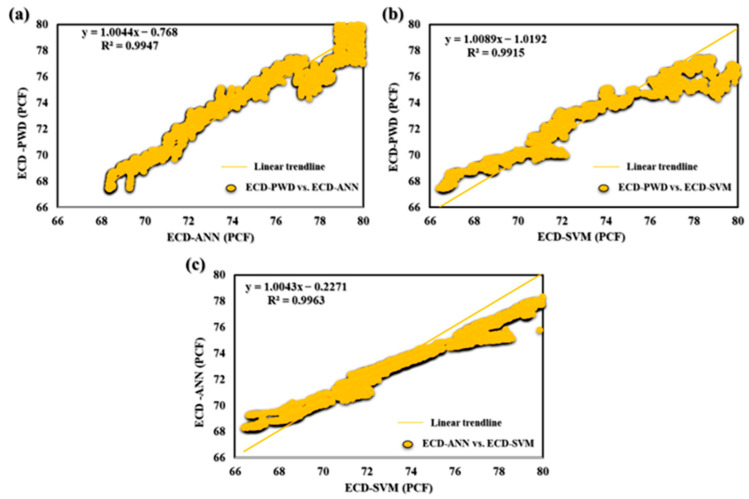
(**a**) ECD-PWD vs. ECD-ANN, (**b**) ECD-PWD vs. ECD-SVM, and (**c**) ECD-ANN vs. ECD-SVM for offshore gas Deviated Well-A.

The subsection presented in this paper allowed for real-time prediction of MW_effc_ and ECD_effc_ while drilling by using AI with the proposed drilling controlling automation for maximizing drilling efficiency. Figure 9 shows how the AI ANN and SVM models for predicting ECD-PWD (PCF) versus depth compare to one another. The findings show that the predicted values and actual measurements match well together, proving the correctness and potency of both models. This comparison offers insightful information about the performance of the two models that may be applied to drilling operations optimization and improved oil and gas production efficiency. Moreover, in Figure 10a–c, the accuracy of the ECD-PWD (PCF) prediction using three different models, namely, ANN, SVM, and ANN and SVM together, is presented. The results showed that the accuracy of the ANN model was 99.47%, while the SVM model achieved an accuracy of 99.15%. The ANN and SVM models had the highest accuracy at 99.63%. These findings suggest that the ANN and SVM model may be the most effective approach for predicting ECD-PWD (PCF) with a high degree of accuracy.

**Figure 11:**
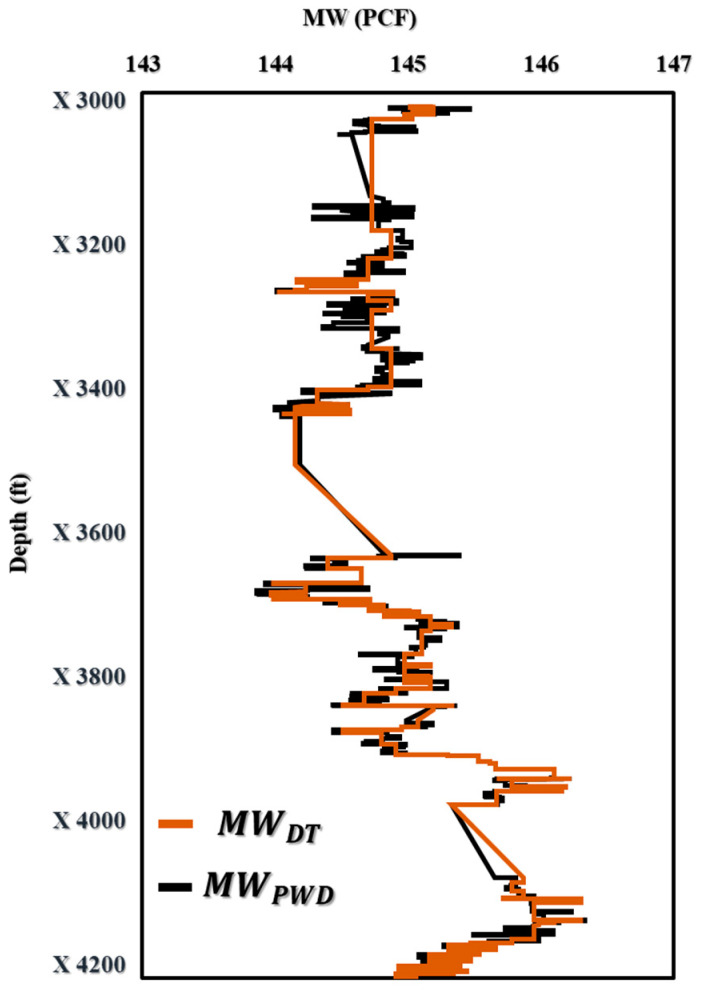
The prediction of MW (PCF) vs. Depth (ft) for offshore gas Deviated Well-A.

For the MW-PWD in Figure 11, the results proved a good fit between the predicted values and the actual measurements, demonstrating the accuracy and effectiveness of the DT model.

**Figure 12:**
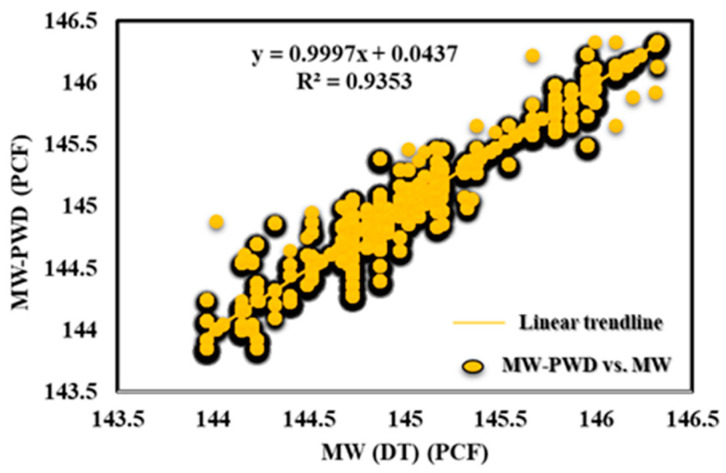
MW-PWD vs. MW (DT) for offshore gas Deviated Well-A.

Figure 11 displays the outcomes of the DT-prediction-model-based MW-PWD. An excellent match may be shown when comparing projected values to actual data, proving the DT model’s accuracy and potency. This result is important because it offers insightful information on the DT model’s performance, which can be utilized to optimize drilling operations and boost the effectiveness of oil and gas drilling performance. Figure 12 shows that the DT model was accurate in predicting mud weight with R^2^ values of 0.94. The bias and accuracy of the mud weight prediction model were found to be 99.86%.

### 3.3. Comparison of Field Applications with AI Applications Using the Novel Models ECD_effc.m_ and MW_effc.m_

**Figure 13:**
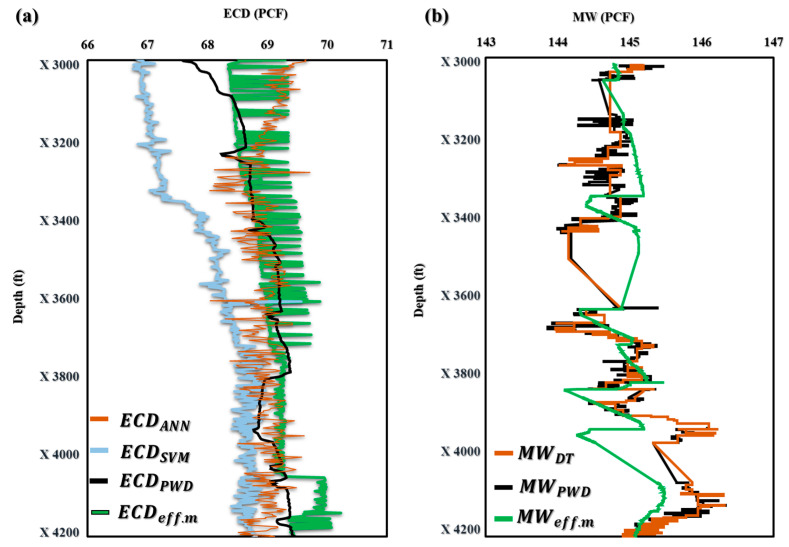
Comparison of field applications with AI applications using the novel models ECD_effc.m_ (**a**) and MW_effc.m_ (**b**).

The comparison of field applications with AI applications using the novel models ECD_effc.m_ and MW_effc.m_ showed the applicability in maximizing the drilling efficiency. As seen in Figure 13a,b, the novel models ECD_effc.m_ and MW_effc.m_ provide highly accurate results that closely match both the AI applications and field measurements (PWD). These findings demonstrate the effectiveness of these models in real-time drilling operations, where accurate predictions are essential for optimizing drilling efficiency and safety. The successful implementation of these models can lead to significant cost savings and improved performance in the oil and gas industry. Furthermore, the comparison between the novel models ECD_effc.m_ and MW_effc.m_ and AI applications has shown that the former are even more accurate. This is because AI applications require input data to predict the output, which can be either ECD or MW. In contrast, the novel models ECD_effc.m_ and MW_effc.m_ are designed to directly provide these outputs, eliminating the need for additional data inputs and improving the accuracy of the results. This advantage allows these models to provide more precise and reliable predictions, making them valuable tools for optimizing drilling operations and improving the efficiency of oil and gas drilling performance.

## 4. The Importance of Predicting the ECD in Real Time

The accuracy and efficiency of drilling operations are enhanced by real-time ECD prediction. If the ECD goes beyond a certain threshold, it may harm the formation, induce wellbore instability, or even result in well control problems such as kicks or blowouts. Drilling engineers and operators can make quick judgements with the aid of real-time ECD monitoring to avert these problems and expensive downtime. Additionally, real-time ECD prediction can aid in improving drilling efficiency. Drilling engineers can maintain ECD within the appropriate range by adjusting drilling parameters such as drilling fluid flow rate and density by continuously monitoring ECD, which leads to enhanced hole-cleaning efficiency and can lessen the chance of formation damage, shorten drilling time, and improve drilling efficiency. As an example, the efficiency of hole cleaning can be evaluated in real time by utilizing a novel model to predict certain parameters by [5]. This real-time prediction can help prevent issues such as high drag, torque, and pipe sticking. By predicting the hole-cleaning index in real time using the developed model, the efficiency of the hole-cleaning index (HCI) can be determined in a straightforward manner [5]:

(24) $$HCI=\frac{K_{m}\cdot {AV}_{m}\cdot EMW}{5867}$$

where $K_{m}$ is the modified consistency index, EMW is the equivalent mud weight, and ${AV}_{m}$ is the modified annulus velocity.

**Figure 14:**
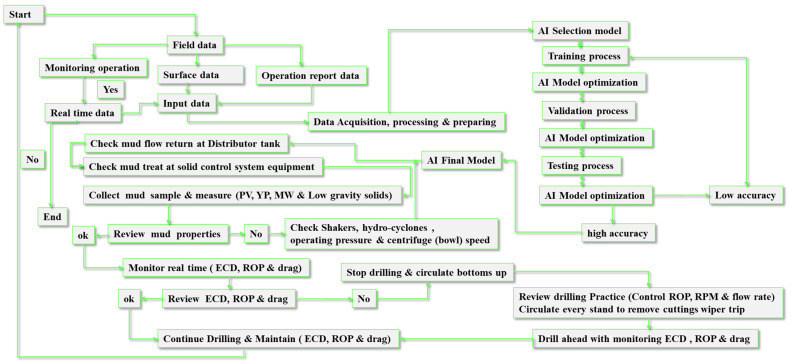
The automated process of utilizing field data to predict ECD by applying AI tools for optimizing the drilling performance efficiency.

Figure 14 shows the automated process of utilizing field data to predict ECD by applying AI tools for optimizing the drilling performance efficiency. As seen in Figure 14, the novel model is able to predict a number of variables that influence drilling operations, such as hole-cleaning effectiveness, cutting concentration, and drilling issues. Current models cannot forecast drilling parameters in real time because they rely on laboratory data. Real-time forecasts may be produced using the established model every second and documented with good depth, enabling early identification and mitigation of any abnormalities. This may lower drilling expenses and reduce the operating time. More importantly, as a result of the work, the flowchart, which is composed of field data that consists of real-time, surface, and operational data, shows the automated process of utilizing field data to predict ECD by applying AI tools, thus enhancing the drilling performance efficiency.

## 5. Conclusions

In this paper, the novel models ECD_effc.m_ and MW_effc.m_ were developed to consider various real-time drilling parameters to optimize drilling performance. These models provide accurate assessments of drilling conditions, identify potential issues in real time, and help prevent complications such as blowouts and stuck pipe incidents. The methodology of the two novel models and the AI application used in this paper can be used to optimize drilling operations, minimize the risks associated with drilling, and maximize drilling efficiency. The following points can be summarized:

1. The field testing and validation of the two novel models, ECD_effc.m_ and MW_effc.m_, have demonstrated their effectiveness in enhancing the efficiency of drilling wells. The results confirm that the use of these models can greatly improve drilling practices and reduce the risk of issues caused by ineffective drilling performance. Furthermore, the automation of these models can further enhance their accuracy and efficiency, optimize drilling operations, and improve safety. In addition, the average accuracy statistical measures for the novel model ECD_eff.m_ were 0.06% and 0.29% for MAE and MSE, respectively. For MW_effc.m,_ the MAE and MSE were 0.11% and 0.08%, respectively.
2. The application of ANN and SVM allowed for the estimation of ECD with a high correlation coefficient of 0.9947 and an average absolute percentage error of 0.23%. Similarly, the application of DT enabled the estimation of MW, achieving a correlation coefficient of 0.9353 and an average absolute percentage error of 1.66%. These models can be used in well design and while drilling to select and adjust the appropriate MW and ECD, eliminating the need for expensive downhole equipment and commercial software and adding excessive chemical additives.
3. The novel models ECD_effc_._m_ and MW_effc_._m_ have been shown to be more accurate than AI applications in predicting ECD_effc_ and MW_effc_. Unlike AI applications, these models directly provide the desired outputs without the need for additional data inputs and PWD, resulting in more precise and reliable predictions. As a result, these models can be valuable tools in optimizing drilling operations and improving the efficiency and safety of oil and gas drilling wells. Overall, this comparison provides valuable insights into the performance of the novel models and their potential benefits for the industry.

## Figures and Tables

**Table 1:** Literature review related to borehole prediction.

No.	Outcomes Measured	Summary	Ref.
1	Pressure	The pressure-while-drilling measurement in real time can assist to avoid some drilling problems.	[25]
2	Dynamic viscosity Density	The dynamic viscosity and density of the ice-chips and drilling-fluid mixture are required to calculate circulation parameters.	[26]
3	ECD Cuttings transport	High equivalent circulating density may cause serious drilling problems in extended-reach drilling.	[27]
4	Risk of differential pressure sticking and drilling mud leakage in reservoir and cap formation	The risk of differential pressure sticking and drilling mud leakage in reservoir and cap formation were both increased in depleted oilfields drilling.	[28]
5	ECD Temperature Wellbore pressure	The geothermal gradient and flow rate were the most influential parameters on the temperature and ECD distribution in the wellbore of the first medium-deep geothermal well.	[29]
6	Equivalent circulating density	The wellbore trajectory may have a major impact on well design.	[30]
7	N/G	Applied managed pressure drilling (MPD) to reach the target depth in tight gas reservoir by zero nonproductive time (NPT).	[31]
8	Fracture pressure	Implement fracture pressure model and design the ECD to avoid the well control problems and lost materials.	[32]
9	Formation fluid Formation collapse Fracture pressure	Utilized pressure while drilling (PWD) to reduce drilling risk by maintaining the ECD and mud weight in the safe zone.	[33]
10	ROP Concentration of cuttings Mud flowrate	The ROP, concentration of cuttings, and flowrate have influenced the ECD and pressure loss.	[13]
11	Newtonian fluid Non-Newtonian fluid	The study was examined using the Couette viscometer, pipe viscometer, and mathematical/physical/AI based on marsh funnel and acoustic technology to estimate the real-time rheological properties of drilling fluid.	[15]
12	Density Temperature Pressure	Predicting the density of drilling fluid by using some computer-based calculations.	[34]

**Table 2:** Summary of recent studies using AI as a prediction tool.

	Input Parameter	Model Used	Data	The Average Absolute Percentage Error	R^2^	Ref.
1	- PV - YP - AV	- ANN	9000	Ranges from 1–5 of 60	N/G	[35]
2	- GPM - ROP - RPM - SPP - WOB	- ANN - ANFIS	3570	- 0.30% - 0.69%	- 0.98 - 0.96	[38]
3	- YP - PV - AV - Flow behavior index - Consistency index	- ANN	1200	- Less than 8%	- 0.96	[7]
4	- Pressure - Temperature	- SVM - FN - RF	1152	- 0.23 - 0.42 - 0.35	- 0.99 - 0.99 - 0.95	[40]
5	- PV - AV - YP - Flow behavior index - Consistency index	- ANN	3000	Less than 6%	-	[36]
6	- Flow rate - Mud weight - PV - YP - TFA - RPM - WOB	- ANN	2000	N/A	- 0.982	[37]
7	- Pressure - Density - Temperature	- PSO-ANFIS - ANFIS - LSSVM	N/G	- N/G - 35.002 - 0.000145	- 0.869 - 0.8502 - 0.999	[39]

**Table 3:** An overview of the kinds of information typically gathered by surface and downhole sensors.

Type of Sensor	Surface Data	Downhole Data
Mud sensor	Pit volume Mud temperature Mud pressure Mud weight Pump strokes	N/A
Well sensor	Temperature Pressure Gas measurements	Temperature Pressure
Directional sensor	N/A	Inclination
Drilling mechanics	RPM Weight on bit Torque Bending moment Rotary torque Hook load Rate of Penetration	RPM Weight on bit Torque on bit Bending moment Downhole vibration
Geological sensor	Cuttings analysis	Density porosity Resistivity Gamma

**Table 4:** The accuracy statistical measures for the novel models MW_effc.m_ and ECD_effc.m_.

Well	Hole Section Size	Hole Section Type	Mud Type	MAE (MW_eff.m_) (%)	MSE (MW_eff.m_) (%)	MAE (ECD_eff.m_) (%)	MSE (ECD_eff.m_) (%)
**A**	12	Deviated	OBM	0.09	0.04	0.05	0.09
**B**	12	Deviated	OBM	0.18	0.15	0.07	0.50
**C**	8 1/2	Horizontal	OBM	0.06	0.04	0.06	0.27
**AVE**				0.11	0.08	0.06	0.29

## Data Availability

The data presented in this study are available on request from the corresponding author. The data are not publicly available due to the internal law of the Saudi Aramco company, “On Export Control”.

## Nomenclature

R3	3 reading revolutions per minutes, cP
R300	300 reading revolutions per minutes, cP
R6	6 reading revolutions per minutes, cP
R600	600 reading revolutions per minutes, cP
ABR-DT	adaptive boosting regressor with decision tree
PSO-ANFIS	adaptive neuro-fuzzy inference system optimized by coupled simulated annealing
ANFIS	adaptive neuro-fuzzy interference system
${CCA}_{2}$	annular concentration based on preconnection circulation period
${CCA}_{1}$	annular concentration based on the volumetric rate of cutting entering the annulus
CRF	circulation rate factor based on circulation and rotation influence
AFPL	annular frictional pressure loss, psi
V_ann_	annular velocity, ft/min
$M_{App}$	apparent viscosity, cP
AI	artificial intelligence
ANN	artificial neural network
${\;V}_{sa}\;$	average slip velocity of drilling cutting, ft/min
α	borehole angle of inclinations, degrees
β	borehole azimuth, degrees
OH	borehole diameter, inch
${ID}_{csg}$	casing inner size, inch
CCA or ${CCA}_{API}$	concentration of cuttings in the annulus
K	consistency factor, cP
${Depth}_{x}$	current measured hole depth, ft
${SPP}_{x}$	current stand pipe pressure, psi
Vsc	cutting slip velocity, ft/min
DT	decision tree
${OD}_{pipe}$ or OD	drill pipe’s outer diameter, inch
${MW}_{eff}$	effective mud weight, pcf
$M_{eff}$	effective viscosity, cP
ECD	equivalent circulating density, pcf
${SPP}_{1}$	first stand pipe pressure when ROP > 1, psi
n	flow behavior index
FN	functional network
GBDT	gradient boost decision tree
HCI	hole-cleaning index
HPHT	high pressure and high temperature
$\alpha_{m}$	hole geometry factor
$\alpha_{m.c}$	hole geometry factor based on the CCA
KNN	k-nearest neighbors
LSSVM	least square support vector machine
${Line}_{ID}$	liner inner diameter
LWD	logging while drilling
LSYP	low shear yield point
MPD	managed pressure drilling
MSE	mean squared error
MWD	measurement while drilling
V_ann_._m_	modified annular velocity, ft/min
V_sar_	the redial cuttings slip velocity
$V_{saa}$	the axial cutting slip velocity
$k_{m}$	modified consistency factor, cP
dc_m_	modified cutting diameter, inch
${ECD}_{effc.m}$	modified equivalent circulating density, pcf
$n_{m}$	modified flow behavior index
${MW}_{effc.m}$	modified mud weight effective, pcf
PV_m_	modified plastic viscosity, cP
YP_m_	modified yield point, cP
$F_{m}$	mud weight affecting the speed of slipping
MW	mud weight, pcf
NPT	nonproductive time
${OD}_{c}$	outer diameter of drill collar, inch
PV	plastic viscosity, cP
PWD	pressure while drilling
GPM	pump flow rate, gal/min
RBF	radial basis function
RF	random forest
ROP	rate of penetration, ft/hr
ReLU	rectified linear unit
x	revolution per gallon ratio
RPM	revolution per minute, rev/min
SPP	stand pipe pressure, psi
${Depth}_{1}$	start measured hole depth, ft
SVM	support vector machine
$V_{ann.dc}$	the annular velocity across drill collar
$V_{ann.dp}$	the annular velocity across drill pipe
CRF	the circulation and rotation influence
R^2^	the coefficient of determination
L_2_	the label column in a dataset
MAE	the mean absolute error
${CCA}_{am}$	the modified average concentration of cuttings in the annulus
$d_{cm}$	the modified cutting diameter
$V_{s.m}$	the average slip velocity with considering the mud weight, ft/min
$V_{s.mn}$	the new average slip velocity with considering the mud weight, ft/min
Qc & QA	the quality control and quality assurance
TR	the transport ratio
$T_{C}$	time for making the connection, min
TFA	total flow area, inch
V_sc_	velocity of cutting slip due to ROP, ft/min
V_s1_ and V_s2_	velocity with consideration for the effective viscosity and apparent viscosity of a fluid, as well as the weight and diameter of the cuttings present in the fluid, ft/min
VSP	vertical seismic profile
Vc	volumetric rate of cutting entering the annulus, fpm
$V_{c}$	volumetric rate of cuttings entering the annulus, ft/min
$W_{c}$	weigh of the cuttings, lb/cf
WOB	weight on bit, KIb
YP	yield point, cP
